# A validation study of the Hospital Anxiety and Depression Scale (HADS) in a large sample of French employees

**DOI:** 10.1186/s12888-014-0354-0

**Published:** 2014-12-16

**Authors:** Christine Bocéréan, Emilie Dupret

**Affiliations:** Department of Psychology, Lorraine University, Bld Albert 1er, 54000 Nancy, France; Psychologist, associated researcher, Cabinet Préventis, Centre d’intervention pour la santé au travail, 91 rue Taitbout, 75009 Paris, France

**Keywords:** Hospital Anxiety and Depression Scale (HADS), Validation study, Employees

## Abstract

**Background:**

The Hospital Anxiety and Depression Scale (HADS) is a questionnaire widely used for detecting anxiety and depressive disorders. It is used extensively in France, but has never been the subject of a full study in a population at work. The objectives of this study were to present some psychometric properties of the HADS on a large sample of French employees.

**Method:**

The HADS questionnaire was given to salaried employees at 19 major French companies as part of their biennial occupational medical examination. In 2011, 20992 employees filled in the questionnaire. HADS’s structure was studied first by exploratory, then confirmatory factorial analyses.

**Results:**

The model selected was the original two-factor structure. The two subscales showed good internal consistency. Women scored higher than the men for anxiety and depression; the scores increased with age; engineers and managers had lower average scores than other occupational status (blue- or white-collar workers and technicians).

**Conclusion:**

The results of the analyses are consistent with those in literature relating to other populations studied in other countries. The HADS questionnaire is pertinent for detecting symptoms of anxiety and depression in a population of people at work.

## Background

The publication of the Hospital Anxiety and Depression Scale (HADS) in 1983 [1] triggered off a large number of significant research studies. There is therefore an abundant literature on the subject. This tool has been translated and validated in many countries and its capacity to detect anxiety and depressive disorders is widely recognized. Nonetheless, as soon as it was published, researchers rightly questioned the structure of the questionnaire, its links with the other tools for determining anxiety and depression and its psychometric properties in general. In the first instance, we shall present the points on which there seems to be a consensus, followed by the one for which this is much less the case.

HADS comprises 14 items, seven of which relate to anxiety symptoms and seven to depressive symptoms. Each item is coded from 0 to 3. The scores for anxiety and depression can therefore vary from 0 to 21, depending on the presence and severity of the symptoms. The authors [1] have proposed cut-off points or thresholds: a score between 0 and 7 does not indicate the presence of the symptoms of anxiety or depression; a score between 8 and 10 indicates the presence of the symptomology but to a moderate degree, therefore doubtful cases; a score greater than or equal to 11 indicates a significant number of symptoms of anxiety or depression corresponding to confirmed cases. The studies concerning the accuracy of these thresholds all showed them to be reliable: the review of the literature by Bjelland et al. [2], covering 747 papers, concluded that the threshold of 8 is decisive in order to not exclude people suffering from anxiety or depressive disorders, which would be the case if the threshold of 10 alone was taken into account. Since 2002, further studies have measured the sensitivity and specificity of the HADS: This tool was proposed in Norway to a general population in the doctor's consulting room [3], in England with people suffering from heart disease [4] and to a representative sample of the German population [5], the threshold of 8 is highly recommended for each of the two scales. More recently, Roberge et al. [6] calculated different thresholds relating to each scale: 10 or more for the anxiety scale, 7 or more for the depression scale. When the HADS is considered on one scale only, a possibility envisaged but not recommended by Zigmond and Snaith [1] as a measure of the intensity of “emotional distress”, the recommended threshold is 13 [5], even 16 [6].

The concurrent validity of each of the two HADS scales, HADS Depression subscale (HADS-D) and HADS Anxiety subscale (HADS-A), has been demonstrated with populations suffering from various pathologies, in different countries and with a variety of methodologies: Clinical interviews and/or standardized tools. Thus, high correlations were found between the scores on the Beck Depression Inventory and the HAD-D scores in diabetic patients [7], in elderly people in hospital, people attending hospital consultations and people in the general population [8] or in patients suffering from cancer [9], to quote only the most recent studies. The Depression Screening Questionnaire has also been used to check the concurrent validity of the HADS-D scale in the patients of general practices [3], and the Quality of Life Questionnaire in Iranian patients suffering from different stages of cancer [10] or people suffering from musculoskeletal disease [11]. Regarding anxiety, the two principal questionnaires used are the State-Trait Anxiety Inventory and the Generalized Anxiety Questionnaire [3,8].

However, the HADS-D and HADS-A are not independent: according to Bjelland et al. [2], the average correlation would be .56, which is not astonishing, given the comorbidity between anxiety and depressive pathologies. The main symptom measured by the HADS-D relates to an anhedonic depression. So, the correlation between sub-score is estimated, in the general population, at between .5 and .6 [12] and is clearly higher in the populations suffering from pathologies, which the vast majority of the studies involved. For this reason, some authors recommend the use of HADS-A to assess both anxious and depressive symptoms [7,13]. It should, however, be remembered that the meta analysis produced by Mitchell, Meader and Symonds [13] involved only patients suffering from cancer and in palliative care, for whom it can easily be understood that anxiety symptoms are closely intertwined with depressive symptoms; the same argument can be advanced for the study by Sultan, Luminet and Hartemann [7] which concerns diabetics, for whom the depressive experience is highly marked by anxiety. Others, that we have already mentioned, recommend the use of the global scale to assess the intensity of emotional distress (e.g. [5,7,13-16]).

The few divergences we have just mentioned are not, however, subject to significant debate. On the other hand, they are revealing about a point on which the authors are not in agreement, that is to say the factorial structure of the HADS. This has been under discussion for almost 30 years and the debate is still ongoing.

Zigmond and Snaith [1] drew up the HADS with two objectives: i) retain only items “based solely on the psychic symptoms of neurosis” (p. 362), ii) “to define carefully and distinguish between the concepts of anxiety and depression” (id.). This is in order to evaluate the disorders more related to the patients' psychological symptoms rather than physical ones. Thus, the depressive dimension is strongly marked by the measure of anhedonia (five items out of seven), a characteristic symptom of depression, and the anxiety dimension by the measure of feelings of tension, worry, fear and panic (five items out of seven). Nonetheless, some of these ten items can give an account of both an emotional state and a physical state as well as the two supplementary items that complete each of the scales. We can estimate that these items do not fully meet the authors' first objective and we shall see that these are the items that are subject to discussion in the majority of cases.

Although some research studies point to a structure with four factors [17,18] or a single factor [5,7,13-16], the main debate concerns two or three factors (cf. literature review of Cosco et al. [19]). Martin [20] deems that we have to take the negative linguistic form of some items into consideration either as a general factor influencing the factors of anxiety and depression, or as a supplementary factor. Others take the tripartite model of Clark and Watson [21,22] as a basis and obtain a more satisfactory factorial solution by considering three factors: An anxiety factor, a depression factor and a negative emotional or psychomotor agitation factor [14,23-28]. Lastly, a large number of research studies point to the initial two-factor structure of HADS [2,3,7-9,12,13,29-40]. Nonetheless, it is difficult, *a priori*, to adopt a position in favor of one or the other, given the very diverse populations involved in the analyses (witness the debate between Friedmann et al. and Dahl et al. [41] and the different statistical methods used (EFA - with orthogonal or oblique rotation - or CFA). In addition, the study by Straat, Andries van der Ark and Sijtsma [42] shows that the differences obtained by the authors in respect of the dimensional structure of the HADS are essentially due to methodological factors. However, Norton et al. [43] present a meta confirmatory factor analysis by which they tested 10 models; although they prefer the solution with a general distress factor, they conclude that a bifactorial model is acceptable.

Our aim is to present some psychometric properties of the HADS on a large population of French employees and to see whether we obtain the same main results as in the literature. The population in our research is original, since it concerns working employees; it is assumed to be in better health than all the other populations. There is no study relating to this type of population. Also, this questionnaire is very often used by companies in France (we specify the context in the next paragraph) without there being any previous psychometric analyses done on such data.

We shall compare our results with the studies involving a general population only [2,5,23,31,33,34] and with a small number of existing French studies [7,14,26,44-46].

## Methods

### Study population

The data were gathered throughout 2011 in 19 major French companies, some of which are established on different sites, representing a total of 32 different French towns (in the Paris area and the other regions). They come from various business sectors:

- Nuclear (6.3%)
- Telecommunications (17.5%)
- Audiovisual (9.8%)
- Construction (9.7%)
- Pharmaceutical (4.4%)
- Banking (7.2%)
- Cosmetics (6.5%)
- Aeronautical (7.5%)
- Petroleum (14.6%)
- Electronics (8%)
- Other (8.5%)

In 70% of cases, it is company head offices which are concerned, the remaining 30% being production sites. The professions of employees within head offices are relatively similar, even though the companies' businesses are different (e.g. Purchasing/marketing/sales; assistance/secretarial; accounting/management/finance; legal; communications; human resources, etc.). On production sites, jobs differ according to the nature of the business. In the whole of our sample (n = 20992), we counted more than 100 different jobs.

### Study instruments

The HADS comprises fourteen items: seven items measure symptoms of anxiety (HADS-A) and seven items measure symptoms of depression (HADS-D). Each item is coded 0 to 3, which gives a score varying between 0 and 21 for each scale. The version used is the French version introduced by Lepine et al. [45] and used since by Ravazi et al. [14], Friedman et al. [25] or Untas et al. [40], for example.

The HADS was preceded by socio-demographic questions. We kept the variables common to the companies. In this way, we possess information relative to gender, age category and occupational status for all our population.

### Study procedure

In the companies that we work with and in the French legal framework for preventing psychosocial risks, the occupational health service administers questionnaires to all the employees during their medical examination on a dedicated computer. One of the questionnaires is the HADS and employees have the ability to print out their scores; this means that they can discuss them immediately with the doctor.

Other questionnaires are also offered to employees but they differ from company to company: thus, some employees complete the Cohen Perceived Stress Scale [47] in 10 or in 4 items and/or a questionnaire measuring psychosocial risks, the Copenhagen Psychosocial Questionnaire [48]. The execution was computerized and the data collected was fully anonymous.

Once a year, usually, companies ask us for a global analysis, by type, by age, by job, etc. and we report the results to the medical and social partners. We also participate, if the company so wishes, in setting up action plans with groups of employees who obtained the lowest scores.

The employees were free to respond or not, but given the context, more than 95% responded, representing a total 20992 people who fully completed the HADS in 2011.

### Statistical analysis

Exploratory Factor Analyses (EFAs) were carried out on all the participants and on several subgroups on SPSS 18.0. The rotation requested was oblique (oblimin rotation) given that the factors are correlated.

The results of the EFAs show that there are several possible factorial solutions. We tested these models with Confirmatory Factor Analysis (CFA) conducted on Lisrel 8.8. We take as our basis the two recommended indicators [49] (that are Root Mean Square Error of Approximation (RMSEA), and Adjusted Goodness of Fit Index (AGFI) which corrects Goodness of Fit Index (GFI) depending on the number of degrees of freedom. A good fit between the theoretical model and the data should be expressed as an RMSEA lower than .05 [50], or even .08 [51] the AGFI should be greater than .90 [52] or even .95 [49].

We then proceeded to carry out a reliability analysis by measuring the internal consistency using Cronbach's alpha and calculating the scale-item correlations (SPSS 18.0). We checked that the scores obtained were compatible with those of the literature by means of variance analyses (SPSS 18.0) and presented the mean scores by subgroup (gender, age and status). Given the size of the population, analyses are easily significant; we also specify thus the effect sizes (Eta-square, η^2^).

## Results

### Validity analysis: factorial structure

We carry out an EFA on all the subjects that leads to extraction of three factors, which account for 37.6%, 9% and 8% of variance (corresponding eigenvalues were 5.3, 1.3 and 1.05). But according to Costello and Osborne [53], this method often overestimates the number of factors: they thus suggest fixing the number of factors manually and comparing the item loading tables. They also give criteria to choose the “cleanest solution”: i) item loading above .30, ii) no or few item crossloading and iii) no factors with fewer than three items. So we have explored a two- and three-factor solution (cf. Table 1).

**Table 1:** **Loading of HADS Items for the Two**- **and Three**-**factor Model (items crossloading are in bold)**

**HADS Item**		**Two-Factor solution**		**Tree-Factor solution**		
		**F1**	**F2**	**F1**	**F2**	**F3**
Anxiety subscale						
HAD1-A1	I feel tense or “wound up”	.54	-.70	.49	-.68	.31
HAD3-A2	I get a sort of frightened feeling as if something awful is about to happen	.46	-.75	.44	-.76	.08
HAD5-A3	Worrying thoughts go through my mind	.52	-.78	.47	-.77	.27
**HAD7-A4**	**I can sit as ease and feel relaxed**	**.47**	**-.38**	.40	-.28	.73
HAD9-A5	I get a sort of frightened feeling like “butterflies” in the stomach	.42	-.80	.39	-.81	.13
HAD11-A6	I feel restless as I have to be on the move	.19	-.50	.09	-.35	.67
HAD13-A7	I get sudden feelings of panic	.40	-.79	.36	-.79	.15
Depression subscale						
HAD2-D1	I still enjoy the things I used to enjoy	.72	-.33	.73	-.36	.09
HAD4-D2	I can laugh and see the funny side of things	.78	-.46	.78	-.48	.14
HAD6-D3	I feel cheerful	.66	-.46	.65	-.47	.14
**HAD8-D4**	**I feel as if I am slowed down**	**.41**	**-.41**	**.44**	**-.48**	**-.25**
HAD10-D5	I have lost interest in my appearance	.60	-.29	.58	-.31	.08
HAD12-D6	I look forward with enjoyment to things	.80	-.39	.81	-.41	.11
**HAD14-D7**	**I can enjoy a good book or TV program**	.57	-.29	**.53**	-.23	**.51**

Quite similar patterns of results are obtained for all subsets (women, men, and the four age groups) with one exception. In women subset analyses, we find the original two-factor solution of Zigmond and Snaith [1]. In other analyses, differences lie in small variations of loadings which do not alter the overall pattern and HAD7-A4 and HAD8-D4 items load cross the two or three factors in the same way.

The three-factor solution reveals two of three disadvantages quoted by the authors: factor 3 consists only of two items and HAD7-A4 and HAD14-D7 items load cross two or three factors. The two-factor solution presents only one disadvantage: HAD7-A4 and HAD8-D4 items load cross the two factors.

We thus choose the two-factor model. We propose to test four models of CFA which take into account all the possible combinations of HAD7-A4 and HAD14-D7 items belonging to one or other factor (*cf*. Table 2).

**Table 2:** **Description of theoretical models tested by CFA (distribution of the HADS items on the factors); the items that load substantially on two factors are in bold**

**Items**	**Model 1 (original model)**		**Model 2**		**Model 3**		**Model 4**	
	**Fact1**	**Fact2**	**Fact1**	**Fact2**	**Fact1**	**Fact2**	**Fact1**	**Fact2**
HAD2-D1	x		x		x		x	
HAD4-D2	x		x		x		x	
HAD6-D3	x		x		x		x	
**HAD8-D4**	**x**			**x**	**x**			**x**
HAD10-D5	x		x		x		x	
HAD12-D6	x		x		x		x	
HAD14-D7	x		x		x		x	
HAD1-A1		x		x		x		x
HAD3-A2		x		x		x		x
HAD5-A3		x		x		x		x
**HAD7-A4**		**x**	**x**		**x**			**x**
HAD9-A5		x		x		x		x
HAD11-A6		x		x		x		x
HAD13-A7		x		x		x		x

Table 3 shows the indicators associated with each model tested.

**Table 3:** **Adjustment indicators of the different models tested**

	**Χ** ^**2**^ (df), p*	**RMSEA**	**AGFI**
Model 1	7078.8 (76), < .001	.067	.94
Model 2	6923.2 (76), < .001	.066	.94
Model 3	7186.8 (76), < .001	.067	.94
Model 4	6977.8 (76), < .001	.067	.94

*Χ^2^: Chi-squared (number of degrees of freedom), significance threshold.

All the Χ^2^ have a high value and are significant, which is not surprising given the amount of data. There is no model which distinguishes itself in a more favorable way than the other one: every four models quite similar fits. Some authors consider the values of these indices to be satisfactory [52]. Nonetheless, they can be improved by allowing correlations between the error variances between some items and the factors or between items themselves. We choose to improve the model 1 because both two links proposed by the program to lower most the Χ^2^ value are respectively the one between HAD8-D4 item and factor 2 and that between HAD7-A4 item and factor 1.

**Figure 1:**
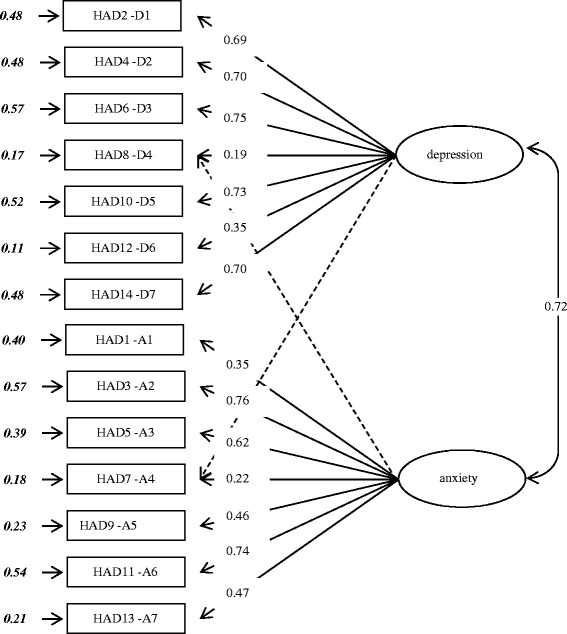
**Factor structure of HADS.** The dotted arrows indicate the correlations recommended by the model for improvement; in bold italics, the r^2^ associated with each scale.

This last analysis shows an X^2^ (74) value of 6372.5 (p < .001), a RMSEA equal to .06 and an AGFI of .96, which almost meets the highest constraints [49,51]. Other numerous links are proposed between items, which would allow us to improve still adjustment indicators. Nevertheless, we prefer to stop there for three reasons: i) the present factorial structure is enough good to report data, ii) it also reports loadings of HAD8-D4 and HAD7-A4 items on factors 1 and 2, as suggested it the EFAs, and iii) we know well that items and factors are inter correlated; it is not thus necessary to weigh down the model by adding links between items only to obtain a RMSEA lower than .05. Figure 1 represents the factor structure of the HADS.

### Reliability analysis: internal consistency

The internal consistency of the two scales is good. Concerning the depression scale, Cronbach's alpha is .78 and the scale/item correlations vary from .54 (HAD8-D4) to .77 (HAD12-D6). Concerning the anxiety scale, Cronbach's alpha is .81 and the scale/item correlations vary from .56 (HAD7-A4 and HAD11-A6) to .78 (HAD5-A3). The anxiety and depression scores are correlated at .62.

### Validity analysis: known groups comparison

The sample is composed of 38.5% of women; the age categories are as follows: 21% of employees are of less than 30 years old, 33.4% from 30 to 39 years old, 28.8% from 40 to 49 years old and 20.8% are more 50 years old. The occupational status categories are as follows: 50.5% are blue- or white-collar Workers and Technicians (WT), 49.5% are middle (engineers or junior executives) or upper Management (top executives) (Ma). The WT have on average a level of study lower than second-year University, Ma have a level of study upper.

Several studies have highlighted the differences in anxiety and/or depression scores depending on gender and age using the HADS [5,26,33,54]. These differences are to be found in the population we used (cf. Table 4). Women have a higher average anxiety score than men (F(1, 20990) = 515.8, p < 0.001, η^2^ = .03); the same is true of the depression score (F(1, 20990) = 32.5, p < 0.001, η^2^ = .002). The anxiety score increases with age (F(3, 20988) = 37.4, p < 0.001, η^2^ = .005) as does the depression score (F(3, 20988) = 128.3, p < 0.001, η^2^ = .02). It should be noted, however, that not all the age groups differ two by two concerning the anxiety score (Bonferroni's Post-Hoc tests): score of employees from 40 to 49 years old does not differ from that of those of more than 50 years old.

**Table 4:** **Mean (M) and Standard Deviation (SD) of the anxiety and depression scores of subgroups**

		**HADS-A**		**HADS-D**	
**Subgroup**		**Mean**	**SD**	**Mean**	**SD**
**Sex**					
	Female	6.55	3.8	3.48	3.2
	n = 8072				
	Male	5.42	3.3	3.22	3.0
	n = 12920				
**Age**					
	Under 30	5.39	3.3	2.64	2.6
	n = 4399				
	30 to 39	5.85	3.5	3.21	3.0
	n = 7008				
	40 to 49	6.06	3.6	3.62	3.3
	n = 5215				
	50 and over	6.09	3.6	3.84	3.4
	n = 4370				
**Occupational status**					
	WT	6.06	3.7	3.65	3.3
	n = 10606				
	Ma	5.65	3.4	2.98	3
	n = 10386				

We also find the expected differences between employees of different occupational status [55,56]: WT have anxiety (F(1, 20990) = 72.3, p < 0.001, η^2^ = .003) and depression scores (F(1, 20990) = 237.2, p < 0.001, η^2^ = .01) significantly higher than managers.

### Average scores according to gender, age group and occupational status

The size of our sample allows us to give indicative scores for the population of French employees according to gender, age and occupational status. Tables 5 and 6 present the average scores of anxiety and depression of the employees according to three variables.

**Table 5:** **Mean (M) and standard deviation (SD) of the anxiety score of subgroups of employees according to gender, age group and occupational status (WT: blue- or white-collar Workers and Technicians; Ma: engineers or junior executives and upper Management)**

**Female M(SD)**			**Male M(SD)**		
**Age according occupational status**			**Age according occupational status**		
Under 30	WT	5.84 (3.6)	Under 30	WT	4.97 (3.1)
		n = 1361			n = 1327
	Ma	5.89 (3.3)		Ma	4.94 (2.8)
		n = 695			n = 1016
30 to 39	WT	6.82 (4)	30 to 39	WT	5.6 (3.4)
		n = 1535			n = 2014
	Ma	6.26 (3.6)		Ma	5.19 (3.1)
		n = 1229			n = 2230
40 to 49	WT	6.97 (3.9)	40 to 49	WT	5.83 (3.4)
		n = 1034			n = 1341
	Ma	6.69 (3.8)		Ma	5.47 (3.2)
		n = 859			n = 1981
50 and over	WT	7.05 (3.9)	50 and over	WT	6.07 (3.4)
		n = 894			n = 1100
	Ma	7.2 (3.8)		Ma	5.38 (3.2)
		n = 465			n = 1911

**Table 6:** **Mean (M) and standard deviation (SD) of the depression score of subgroups of employees according to gender, age group and occupational status (WT: blue- or white-collar Workers and Technicians; Ma: engineers or junior executives and upper Management)**

**Female M(SD)**			**Male M(SD)**		
**Age according occupational status**			**Age according occupational status**		
Under 30	WT	2.88 (2.9)	Under 30	WT	2.74 (2.7)
		n = 1361			n = 1327
	Ma	2.41 (2.5)		Ma	2.34 (2.4)
		n = 695			n = 1016
30 to 39	WT	3.66 (3.2)	30 to 39	WT	3.54 (3.2)
		n = 1535			n = 2014
	Ma	3.05 (3.0)		Ma	2.69 (2.6)
		n = 1229			n = 2230
40 to 49	WT	4.16 (3.6)	40 to 49	WT	4.02 (3.3)
		n = 1034			n = 1341
	Ma	3.61 (3.3)		Ma	3.08 (3.0)
		n = 859			n = 1981
50 and over	WT	4.33 (3.6)	50 and over	WT	4.45 (3.5)
		n = 894			n = 1100
	Ma	3.99 (3.5)		Ma	3.22 (3.1)
		n = 465			n = 1911

## Discussion

After carrying out EFAs on all the people in our sample, as well as the sub-groups defined according to gender and age, we used CFA to test the different possible theoretical models. It turns out that the original model, as defined by Zigmond and Snaith [1] is the one that demonstrates the best fit. This is therefore the one we selected at least for the population we are interested in, that is to say, French employees.

In EFAs, two types of items are observed: items which substantially load one of the two factors and two items with smaller loadings that load the two factors. Thus, HAD2-D1, HAD4-D2, HAD6-D3, HAD10-D5, HAD12-D6 and HAD14-D7 items have a loading that varies from .57 to .80 on factor 1, which we identify as accounting for the scale of depression. Factor 2, corresponding to the anxiety scale, comprises HAD1-A1, HAD3-A2, HAD5-A3, HAD9-A5, HAD11-A6 and HAD13-A7 items whose loadings vary from -.50 to -.80. Two items HAD8-D4 and HAD7-A4 do not clearly load on one of the two factors.

We propose to test four models of CFA: the original model and the three others which take into account all the possible combinations of items HAD8-D4 and HAD7-A4 belonging to one or other factor (*cf*. Table 2). The chosen model is the original two-factor model but, to obtain a good adequacy of the model with the data, we have of to add a link between HAD8-D4 and the anxiety factor as well as a link between HAD7-A4 and depression factor; what was expected according to the results of the EFAs. These items are often quoted in the literature as having no weight satisfying on the factor to which they are supposed to belong (e.g. [8-10,38,39]). Roberge et al. [6] list the various interpretations mentioned by authors in literature (p. 176–177): the item HAD7-A4, in its formulation, refers to the anxious symptomatology “I cannot sit at ease” and to depressive symptoms related to anhedonia ‘I cannot feel relaxed”. As for item HAD8-D4, if questions are asked about the psychic and motor slowing, which may be a symptom of depression, (“I feel as if I am slowed down”), it can also be reminiscent of the anxious inhibition sometimes present in the anxious spectrum [57].

Nevertheless, even if we did not find the “true” original model, we consider that we can continue to base ourselves on the calculation of the scores according to Zigmond and Snaith [1] with seven items relative to the depression and seven items relative to the anxiety. Indeed, there have been a large number of studies concerning the thresholds calculated from the initial anxiety and depression scales. These studies [2-5] have largely validated the use of thresholds (one threshold at 8 and another at 11) and a calling into question of the calculation of anxiety and depression scores necessarily calls into question the value of the thresholds. The last reason is related to our sample: the HADS was devised *a priori* for detecting the presence of anxiety and depressive disorders in hospital patients. Although the two-factor structure was also found in general populations [3,5,8,33,34], our sample is not fully comparable to a general population because it only concerns people at work. However, when we compare groups (according to gender, age, occupational status and especially other variables specific to each company, such as business line or department), using initial anxiety and depression scores or scores based only on the five items that most load the anxiety (HAD1-A1, HAD3-A2, HAD5-A3, HAD9-A5 and HAD13-A7) and depression factors (HAD2-D1, HAD4-D2, HAD6-D3, HAD10-D5 and HAD12-D6), the global results are unchanged: We find exactly the same groups with the highest scores. As the important aspect of our psychosocial risk prevention approach is to detect "at risk" groups, the scores calculated on the initial scales remain valid and we may, in addition, use the thresholds.

We presented the averages of the scores of anxiety and depression by subgroups (by crossing gender, age group and status). We cannot exactly compare these results in detail with the results published in the literature, with the exception of a few studies that validated the questionnaire with a general population [5,34] or included a control group taken from the general population [8,15]. The overall mean values and the mean values by gender (when these are given) are either comparable to those of this study, or slightly higher, as in the case of the German validation study [5] for the depression score; in the latter case, the population was older than the one in our study, which accounts for the differences. Nevertheless, we can note that women have a higher score of anxiety on average than men (6.55 *vs* 5.42) and that the scores of anxiety and especially depression increase with the age (HADS-A: 5.39 for the 30-year-old employees less *vs* 6.09 for those of 50 and more years old; HADS-D: 2.64 for the 30-year-old employees less *vs* 3.84 for those of 50 and more years old). Occupational status also impacts scores: in every age group, blue- or white-collar workers and technicians have anxiety and depression scores higher than those of engineers or junior executives and upper Management. For example, anxiety (depression) mean score for employees from 30 to 39 years old is 6.14 (3.62) for blue- or white-collar workers and technicians *vs* 5.55 (2.83) for engineers or junior executives and upper Management. The influence of the gender can be illustrated by the difference between anxiety scores of women and the men who are more than 50 years old: if we mention these differences with cut-off defined by Zigmond and Snaith [1], 18.4% of women have a score upper to 11 versus 9% of men. Occupational status influence can be illustrated in its turn by the difference between depression scores of the employees who are more than 50 years old: 6.8% of blue- or white-collar workers and technicians have a score upper to 11 versus 3.7% of managers. These results are consistent with those found in literature; we cite in particular Cohidon [57] who reports the results of the SMPG "Mental health in the general population" survey conducted between 1999 and 2003 on a sample of 36,000 people in France. The questionnaire used was the Mini [58]. The author reports that mood disorders affect 11% of men and 16% of women; anxiety disorders 17% of men and 25% of women. Analysis of the data shows equal and systematic differences according to socio-professional category: the least qualified persons are those most affected by disorders. The hypothesis which we are able to put forward (which concerns our results and also Cohidon's) is that persons on a lower socio-economic level have a more restricted access to care, whether in financial terms and/or on a personal level [59,60]. Actually, the general practitioner remains the preferred point of contact for these people, whereas executives and managers are less reluctant to approach a psychiatrist or psychologist (the latter is never covered by health insurance in France).

### Limitations of the study

The way in which these data were gathered has its advantages and disadvantages and determines the limits of this study. Indeed, the circumstances in which they are gathered is the yearly or two-yearly examination by the company doctor. The high rate of participation comes from the fact that people are more convinced of the anonymity of their responses when information is given to the workplace health department. Furthermore, employees have the ability to print out their scores, in order to discuss them with the doctor who, in all cases, addresses the subject with the employee. But the very fact of completing a questionnaire in the workplace can necessarily produce a bias which we can observe, given that all our data are collected in the same context. The employee can attempt to minimize his/her scores for anxiety or depression symptoms, just as he/she can do the opposite. According to the doctors whom we meet regularly, the HADS helps them to detect incipient anxiety or depression problems: an employee who scores higher than 10 or 11 in an interview on one of the two scales present, behavior which will leave no doubt in the doctor's mind on the necessity of asking more detailed questions on personal or work-related problems; an employee with a score of between 8 and 11 will attract the doctor's attention and he/she will go into more detail to determine whether the employee is actually presenting symptoms resulting from anxiety or depression.

From a psychometric point of view, it has not been possible to verify a number of elements. Given that employees' responses are completely anonymous, it is impossible to do a test and retest, to improve the degree of accuracy. Similarly, convergent validity can only be addressed by reports from doctors and their experience. Actually, in this situation, it is not possible to offer employees different questionnaires relating to other measurements for anxiety and depression, so as to test their concurring validity.

Finally, we do not have many independent variables; for example, we would have wished to know the level of studies of our subjects to make comparisons with the literature; we have not the exact age (but only groups of age) and no information on the personal life of the subjects.

## Conclusion

The findings from this study are consistent with the analyses conducted with other populations in other countries: the HADS has good reliability and discriminant validity; its bi-dimensional structure allows one score to be calculated for anxiety symptoms and one score for depression symptoms. However, as Straat, Andries van der Ark and Sijtsma [42] suggest, and given the recurring results for the problem items, perhaps this questionnaire should be reviewed.

However, the HADS remains a useful instrument for detecting anxiety and depression symptoms, both at an individual and a collective level. As we have explained, the individual level is not within our remit but that of the company doctor. On the other hand, we are more particularly interested at a collective level in the context of preventing psychosocial risks. The population covered by this study is obviously not representative of all French employees, since our data practically only concerns major companies. Nevertheless, we update our data every year and we can compare a given company with all the other companies on "our panel", obviously taking into consideration the characteristics of each of them (gender, age, etc.). We therefore see a wide variability between the average scores for anxiety and depression in companies: for example, for 2011 data, the average anxiety score varies between 10.3 (SD = 4.4) and 5.11 (SD = 3.07); the average depression score varies between 6.37 (SD = 4.07) and 2.51 (SD = 2.45). Most of the time, this great variability is due to the economic situation in that business sector; the variability within a company (between different jobs or different departments) results more from internal characteristics (management policy, training, etc.).

The last report drafted by experts and asked by the French Ministry of Employment and Health shows that the anxious and depressive disorders are the ones which are the most present in the population of employees, that they recover from psychosocial risk factors and that it is thus important to measure them [61]. Even if the HADS is criticized [42], it remains a quick and relevant tool for use in companies.

### Ethical approval

French ethics committees, named « Comité de Protection de Personnes » (CCP) are constituted by doctors in medicine, by jurists, by people qualified in biomedical research, by hospitable pharmacists, by competent people as regards questions of ethics and by representatives of associations of user of the health system. They supervise biomedical research and aim for insure at protection of the participants. Law of August 9^th^ 2004 grants them the decision-markers’s role in the authorization of research (Art. L. 1123–6 of Public Health Code). Our ethics committee deemed our study not suitable of ethical review (Art. L. 1123–1 of Public Health Code). A letter from our ethics committe gives evidence of it.

## Abbreviations

HADS: Hospital Anxiety and Depression Scale
HADS-A: HADS Anxiety subscale
HADS-D: HADS depression subscale
EFA: Exploratory factor analysis
SPSS: Statistical package for the Social Sciences
CFA: Confirmatory factor analysis
RMSEA: Root Mean Square Error of Approximation
AGFI: Adjusted Goodness of Fit Index
GFI: Goodness of Fit Index
Χ^2^: Chi squared

## References

[1] Zigmond AS, Snaith P (1983). The hospital anxiety and depression scale. Acta Psychiatr Scand.

[2] Bjelland I, Dahl AA, Haug TT, Neckelmann D (2002). The validation of the Hospital Anxiety and Depression Scale. An updated literature review. J Psychosom Res.

[3] Olssøn I, Mykletun A, Dahl AA (2005). The hospital anxiety and depression rating scale: a cross-sectional study of psychometrics and case finding abilities in general practice. BMC Psychiatry.

[4] Poole NA, Morgan JF (2006). Validity and reliability of the Hospital Anxiety and Depression Scale in a hypertrophic cardiomyopathy clinic: the HADS in a cardiomyopathy population. Gen Hosp Psychiat.

[5] Hinz A, Brähler E (2011). Normative values for the Hospital Anxiety and Depression Scale (HADS) in the general German population. J Psychosom Res.

[6] Roberge P, Doré I, Menear M, Chartrand E, Ciampi A, Duhoux A, Fournier L (2013). A psychometric evaluation of the French Canadian version of the Hospital Anxiety and Depression Scale in a large primary care population. J Affect Disord.

[7] Sultan S, Luminet O, Hartemann A (2010). Cognitive and anxiety symptoms in screening for clinical depression in diabetes. A systematic examination of diagnostic of the HADS and BDI-SF. J Affect Disorders.

[8] Michopoulos I, Douzenis A, Kalkavoura C, Christodoulou C, Michalopoulou P, Kalemi G, Fineti K, Patapis P, Protopapas K, Lykouras L (2008). Hospital Anxiety and Depression Scale (HADS): validation in a Greek general hospital sample. Ann Gen Psychiat.

[9] Muszbek K, Szekely A, Balogh EM, Molnar M, Rohansky M, Ruzsa A, Varga K, Szöllosi M, Vadasz P (2006). Validation of the Hungarian translation of hospital anxiety and depression scale. Qual Life Res.

[10] Montazeri A, Vahdaninia M, Ebrahimi M, Jarvandi S (2003). The Hospital Anxiety and Depression Scale (HADS): translation and validation study of the Iranian version. Health Qual of Life Out.

[11] Härter M, Reuter K, Gross-Hardt K, Bengel J (2001). Screening for anxiety, depressive and somatoform disorders in rehabilitation: validity of HADS and GHQ-12 in patients with musculoskeletal disease. Disabil Rehabil.

[12] Schönberger M, Ponsford J (2010). The factor structure of the Hospital Anxiety and Depression Scale in individuals with traumatic brain injury. Psychiat Res.

[13] Mitchell AJ, Meader N, Symonds P (2010). Diagnostic validity of the Hospital Anxiety and Depression Scale (HADS) in cancer and palliative settings: a meta-analysis. J Affect Disorders.

[14] Razadi D, Delvaux N, Farvacques C, Robaye E (1989). Validation of the French version of the HADS in a population of inpatients with cancer. Rev Psychol Appl.

[15] Herrmann C (1997). International experiences with the Hospital Anxiety and Depression scale - A review of validation data and clinical results. J Psychosom Res.

[16] Krespi Boothby MR, Cases A, Carrington K, Mulholland I, Bolger T (2010). The accuracy of HADS in detecting emotional distress in male prisoners. Procedia S+BS.

[17] Andersson E (1993). The Hospital and Depression Scale. Homogeneity of the subscales. Soc Behav Personal.

[18] Martin CR, Thompson DR (2000). A psychometric evaluation of the Hospital Anxiety and Depression Scale in coronary care patients following acute myocardial infarction. Psychology, Health & Medecine.

[19] Cosco T, Doyle F, Ward M, McGee H (2012). Latent structure of the The Hospital Anxiety and Depression Scale: A 10 year systematic review. J Psychosom Res.

[20] Martin CR (2005). What does the Hospital Anxiety and Depression Scale (HADS) really measure in liaison psychiatry settings?. Curr Psychiat Rev.

[21] Clark LA, Watson D (1991). Tripartite model of anxiety and depression: psychometric evidence and taxonomic implications. J Abnorm Psychol.

[22] Watson D, Clark LA (1995). Testing a tripartite model: II. Exploring the symptom structure of anxiety and depression in student, adult, and patient samples. J Abnorm Psychol.

[23] Leung CM, Ho S, Kan CS, Hung CH, Chen CN (1993). Evaluation of the Chinese version of the Hospital Anxiety and Depression Scale. A cross-cultural perspective. Int J Psychosom.

[24] Dunbar M, Ford G, Hunt K, Der G (2000). A confirmatory factor analysis of the Hospital Anxiety and Depression scale: Comparing empirically and theoretically derived structures. Brit J Clin Psychol.

[25] Friedman S, Samuelian JC, Lancrenon S, Even C, Chiarelli P (2001). Three-dimensional structure of the Hospital Anxiety and Depression Scale in a large French primary care population suffering from major depression. Psychiat Res.

[26] Caci H, Baylé FJ, Mattei V, Dossios C, Robert P, Boyer P (2002). How does the Hospital Anxiety and Depression Scale measure anxiety and depression in healthy subjects?. Psychiat Res.

[27] Martin CR, Lewin RJP, Thompson DR (2003). A confirmatory factor analysis of the Hospital Anxiety and Depression Scale in coronary care patients following acute myocardial infarction. Psychiat Res.

[28] Martin CR, Bonner A, Brook A, Luscombe C (2006). Factor structure and use of the Hospital Anxiety and Depression Scale in the homeless and socially marginalized. Psychology, Health & Medicine.

[29] Moorey S, Greer S, Watson M, Gorman C, Rowden L, Tunmore R, Robertson B, Bliss J (1991). The factor structure and factor stability of the hospital anxiety and depression scale in patients with cancer. Brit J Psychiat.

[30] Spinhoven PH, Ormel J, Sloekers PP, Kempen GIJ, Speckens AE, Van Hermerts AM (1997). A validation study of the Hospital Anxiety and Depression Scale (HADS) in different groups of Dutch subjects. Psychol Med.

[31] Lisspers J, Nygren A, Söderman E (1997). Hospital Anxiety and Depression Scale (HAD): some psychometric data for a Swedish sample. Acta Psychiat Scan.

[32] Bedford A, De Pauw K, Grant E (1997). The structure of the hospital anxiety and depression scale (HAD): an appraisal with normal, psychiatric and medical patient subjects. Pers Indiv Differ.

[33] Crawford JR, Henry JD, Crombie C, Taylor EP (2001). Normative data for the HADS from a large non-clinical sample. Brit J Clin Psychol.

[34] Mykletun A, Stordal E, Dahl AA (2001). Hospital Anxiety and Depression (HAD) scale: factor structure, items analyses and internal consistency in a large population. Brit J Psychiat.

[35] Flint AJ, Rifat SL (2002). Factor structure of the hospital anxiety and depression scale in older patients with major depression. Int J of Geriatr Psych.

[36] Smith AB, Selby PJ, Velikova G, Stark D, Wright EP, Gould A, Cull A (2002). Factor analysis of the Hospital Anxiety and Depression Scale from a large cancer population. Psychol Psychother-T.

[37] Herrero ML, Blanch J, Peri JM, De Pablo J, Pintor L, Bulbena A (2003). A validation study of the hospital anxiety and depression scale (HADS) in a Spanish population. Gen Hosp Psychiat.

[38] Pais-Ribeiro J, Silva I, Ferreira T, Martins A, Meneses R, Baltar M (2007). Validation study of a Portuguese version of the Hospital Anxiety and Depression Scale. Psychology, Health & Medicine.

[39] Gough K, Hudson P (2009). Psychometric properties of the Hospital and Depression Scale in family caregivers of palliative care patients. J Pain Symptom Manag.

[40] Untas A, Aguirrezabal M, Chauveau P, Leguen E, Combe C, Rascle N (2009). Anxiété et dépression en hémodialyse : validation de l'Hospital Anxiety and Depression Scale (HADS). Nephrol Ther.

[41] Friedmann S, Even C, Samuelian JC, Guelfi JD (2002). Factor structure of the Hospital Anxiety and Depression (HAD) scale. Brit J Psychiat.

[42] Straat JH, Van der Ark LA, Sijtsma K (2013). Methodological artifacts in dimensionality assessment of the hospital anxiety and depression scale (HADS). J Psychosom Res.

[43] Norton S, Cosco T, Doyle F, Done J, Sacker A (2013). The Hospital Anxiety and Depression Scale: A meta confirmatory factor analysis. J Psychosom Res.

[44] Lépine JP, Godchau M, Brun P (1985). Anxiety and depression in inpatients. Lancet.

[45] Lepine JP, Godchau M, Brun P, Lemperière T (1985). Evaluation de l'anxiété et de la dépression chez des patients hospitalisés dans un service de médecine interne. Ann Med-Psychol.

[46] Lépine JP, Guelfi JD (1996). L'échelle HAD. L'évaluation clinique standardisée en psychiatrie.

[47] Cohen S, Kamarck T, Mermelstein R (1983). A global measure of perceived stress. J Health Soc Behav.

[48] Dupret E, Bocéréan C, Teherani M, Feltrin M, Petersen J (2012). Psychosocial risks evaluation : French validation of the Copenhagen Psychosocial Questionnaire (COPSOQ). Scand J Public Health.

[49] Hu L, Bentler PM (1999). Cutoff criteria for fit indexes in covariance structure analysis: Conventional criteria versus new alternatives. Struct Equ Modeling.

[50] Steiger JH (1990). Structural model evaluation and modification: an interval estimation approach. Multivar Behav Res.

[51] Browne MW, Cudeck R, Bollen KA, Long JS, Newbury CA (1993). Alternative ways of assessing model fit. Testing structural equation models.

[52] Bentler PM, Bonett DG (1980). Significance tests and goodness-of-fit in the analysis of covariance structures. Psychol Bull.

[53] Costello AB, Osborne J: **Best practices in exploratory factor analysis: four recommendations for getting the most from yours analysis.** *Pract Asses Res & Eval* 2005, **10**(7). Available online: http://pareonline.net/getvn.asp?v=10&n=7.

[54] Stordal E, Bjartveit Krüger M, Dahl NH, Mykletun A, Dahl AA (2001). Depression in relation to age and gender in the general population: the Nord-Trøndelag Health Study (HUNT). Acta Psychiat Scan.

[55] Stellman J (2000). Encyclopédie de sécurité et de santé au travail.

[56] Besançon G (2005). Manuel de Psychopathologie.

[57] Cohidon C (2007). Prévalence des troubles de santé mentale et conséquences sur l’activité professionnelle en France dans l’enquête « Santé mentale en population générale ». Bulletin épidémiologique hebdomadaire.

[58] Sheehan DV, Lecrubier Y, Sheehan KH, Amorim P, Janavs J, Weiller E, Hergueta T, Baker R, Dunbar GC (1998). The Mini-International Neuropsychiatric Interview (M.I.N.I.): the development and validation of a structured diagnostic psychiatric interview for DSM-IV and ICD-10. J Clin Psychiatry.

[59] Cases C, Salines E (2004). Statistiques en psychiatrie en France: données de cadrage. Revue Française des Affaires Sociales.

[60] Legros M, Bauer D, Goyaux D (2012). Pour un accès aux soins plus égal et facilité à la santé et aux soins.

[61] Askenazy P, Baudelot C, Brochard P, Brun JP, Cases C, Davezie P, Falissard B, Gallie D, Gollac M, Griffiths A, Grignon M, Imbernon E, Leclerc A, Molinier P, Niedhammer I, Parent-Thirion A, Verger D, Vézina M, Volkoff S, Weill-Fassina A (2011). Mesurer les facteurs psychosociaux de risque au travail pour les maîtriser. ᅟ.

